# Investigation of the genetic causes of non-syndromic hearing loss in the Western region of Saudi Arabia

**DOI:** 10.1186/1471-2164-15-S2-P50

**Published:** 2014-04-02

**Authors:** Kamal Daghistani, Angham Abdulkarim, Afaf Bamanie, Malek Safieh, Samira Sogaty, Abdulmonem Al-Shaikh, Khalid O Abulnaja, Manar Ata, Ashraf Dallol

**Affiliations:** 1 Center of Excellence in Genomic Medicine Research, King Abdulaziz University, Jeddah, KSA; 2 Department of Audiology, King Abdulaziz University Hospital, King Abdulaziz University, Jeddah, KSA; 3 Jeddah Institute for Speech and Hearing (JISH), Jeddah, KSA; 4 Department of Genetic Diseases, King Fahd General Hospital, Jeddah, KSA; 5 Department of Otolaryngology, King Fahd General Hospital, Jeddah, KSA; 6 Department of Biochemistry, King Abdulaziz University, Jeddah, KSA

## Background

For every thousand children born in the world, there is one who is born with a hereditary form of hearing loss. In Saudi Arabia, the rate of children affected with sensineuronal hearing loss (SNHL) was estimated to be approximately 26 children out of 1000 [1]. Alone in the western region (Jeddah, Makkah, Al-Taif) of the country, there are over 1350 students at special schools for the hearing impaired. Provision of specialized care and education to those children require a significant amount of resources and dedication. In addition, the social stigma usually associated with a deaf child may affect his or her response to treatment or care. The serious problem caused by hearing impairment in Saudi Arabia has been recognized for over two decades. However, this is not reflected in the amount of studies at the genetic basis of it. There is almost complete lack of knowledge on the genetic basis of deafness in the Kingdom is preventing the provision of world-class genetic counseling to the parents of the affected children who in large accept their "fate" but they often have one question on their minds: "Will my next child be the same?” In addition, prevention in the form of pre-marital screening of carriers (potentially considering the high rate of consanguineous marriages) is currently impossible.

## Materials and methods

Using a combination of targeted linkage analysis and PCR-sequencing that allows analyzing up to 10 genetic loci associated with SNHL.

## Results

Our results showed excluding LRTOMT, TMHS and TMIE as major genetic loci underlining SNHL in a cohort of 100 patients from the Western region of Saudi Arabia.

## Conclusions

As there are over 60 genetic loci responsible of SNHL, traditional methods will prove cumbersome and time-consuming exercise. We are currently applying whole-exome sequencing in order to identify the genetic causes of SNHL.

The authors would like to thank the KACST, Riyadh, Saudi Arabia for funding this research Project (ID: ARP-30-304).
